# Integrative Gene Network Construction to Analyze Cancer Recurrence Using Semi-Supervised Learning

**DOI:** 10.1371/journal.pone.0086309

**Published:** 2014-01-31

**Authors:** Chihyun Park, Jaegyoon Ahn, Hyunjin Kim, Sanghyun Park

**Affiliations:** Department of Computer Science, Yonsei University, Seoul, South Korea; Semmelweis University, Hungary

## Abstract

**Background:**

The prognosis of cancer recurrence is an important research area in bioinformatics and is challenging due to the small sample sizes compared to the vast number of genes. There have been several attempts to predict cancer recurrence. Most studies employed a supervised approach, which uses only a few labeled samples. Semi-supervised learning can be a great alternative to solve this problem. There have been few attempts based on manifold assumptions to reveal the detailed roles of identified cancer genes in recurrence.

**Results:**

In order to predict cancer recurrence, we proposed a novel semi-supervised learning algorithm based on a graph regularization approach. We transformed the gene expression data into a graph structure for semi-supervised learning and integrated protein interaction data with the gene expression data to select functionally-related gene pairs. Then, we predicted the recurrence of cancer by applying a regularization approach to the constructed graph containing both labeled and unlabeled nodes.

**Conclusions:**

The average improvement rate of accuracy for three different cancer datasets was 24.9% compared to existing supervised and semi-supervised methods. We performed functional enrichment on the gene networks used for learning. We identified that those gene networks are significantly associated with cancer-recurrence-related biological functions. Our algorithm was developed with standard C++ and is available in Linux and MS Windows formats in the STL library. The executable program is freely available at: http://embio.yonsei.ac.kr/~Park/ssl.php.

## Introduction

Identifying cancer biomarkers for diagnosis and prognosis is one of the most important research fields in bioinformatics. The use of accurate cancer biomarkers can help to determine the appropriate therapy based on patient status. These biomarkers can be presented as a list of genes or gene network structure. Microarray based gene expression has been used to identify these biomarkers [1], [2], [3]. In addition, several recent studies have used not only gene expression data, but also interactome data to enhance the predictive performance. Known cancer related genes are not distinguishable by gene expression level alone. Chuang *et al*. demonstrated that the integration of interactome and transcriptome data was useful for the identification of coexpressed functional sub-networks, and the interactions of the sub-networks acted as a marker with higher classification accuracy [4]. Taylor *et al*. analyzed global modularity in protein interaction networks and revealed that the intermodular hub, one of two types of hubs, was more frequently associated with oncogenesis [5]. Ahn *et al*. proposed a novel and accurate classification method using integration of both interactome and transcriptome data [6]. They also constructed cancer-specific gene networks derived from their classification method and revealed that cancer-related genes in a network play an important role in cancer [6].

Although gene expression and interactome data are very useful for cancer research, the relatively small number of samples compared to the number of genes leads to challenges in analysis [7]. The reliability of discovering genes differentially expressed in two different conditions is decreased by small sample sizes. There have been attempts to overcome this limitation of microarray-based gene expression data [8]. Shi *et al*. mentioned that obtaining microarray data with clinical follow-up information is time consuming, expensive, and limited by sample availability [9]. These findings imply that the existing supervised-learning-based approaches that only use labeled data still have limitations.

One approach to supplementing the small quantities of labeled data is semi-supervised learning, which is a combination of super-vised and unsupervised methods. Semi-supervised learning combines labeled and unlabeled data to construct a learning model with improved accuracy [10]. Generally, semi-supervised classification is used when there are more unlabeled data than labeled data. In such a case, it is thought that the knowledge of the unlabeled data will be useful in the inference of accurate classification rules during the learning process.

Recently, semi-supervised learning based approaches have been widely applied to biological data analysis including genetic interactions. You *et al*. developed a graph-based semi-supervised learning classifier that can predict pairwise synthetic genetic interactions [11]. Because genetic interaction profiles can help create a better understanding of the linkages between genes and functional pathways, an accurate algorithm to predict genetic interactions is highly desirable despite the lack of a high precision functional gene network. Semi-supervised learning approaches have also been applied to prognosis related studies. Nguyen *et al*. proposed a semi-supervised learning based method to predict genes involved in disease by inferring both disease genes and their neighbors through protein interaction networks [12]. Bair *et al*. proposed using both available clinical data and gene expression data to identify the subset of the genes used to perform semi-supervised clustering [13]. Their method was used to reveal subtypes of cancer and predict patient survival. Joshua Smith *et al*. used gene expression profiles to identify a gene classifier associated with a high risk of metastasis and death from colon cancer [14].

As mentioned above, semi-supervised approaches can supplement the limitations of gene expression data analysis, such as lack of an assigned clinical class for each patient. Shi *et al*. proposed a semi-supervised classifier based on low density separation that can identify high-risk and low-risk patients [9]. That study, which used labeled and unlabeled gene expression samples, showed enhanced accuracy compared with existing approaches based on supervised learning. However, there has not been an attempt to apply both semi-supervised learning and the integration of interactome and transcriptome data to overcome the small number of labeled samples and to improve the performance of classification and prediction. The integration of heterogeneous data can help to distinguish more significant genes from the gene expression data used to build classifiers, as mentioned above.

In this article, we used graph regularization and integration of transcriptome and interactome data to build a novel semi-supervised learning-based classifier for human cancer, and constructed a cancer-specific gene network. The graph regularization is based on the ‘manifold assumption,’ where the construction of graph models is an important phase. In design of the graph model for classification, we constructed the graph using labeled and unlabeled samples as nodes. The connection between two samples was calculated using the selected informative gene pairs. In selecting useful gene pairs, we integrated Protein-Protein Interaction (PPI) data with gene expression data. PPI data provided information about the functional relationship among proteins and was applied to genes connected by PPIs. After selecting gene pairs, we applied a scoring scheme proposed in a previous paper [6]. We focused on breast, colorectal, and prostate cancers to predict cancer relapse. Three cancer patients’ mRNA expression data included both unlabeled and labeled samples.

We demonstrated that (i) the proposed semi-supervised learning based classification enhanced prediction performance compared with existing methods, including TSVM, which is a semi-supervised learning version of SVM, (ii) the proposed method was applicable to different cancers, (iii) the proposed method was robust regardless of the class label ratio and (iv) the cancer-specific gene network derived from the classifier was biologically meaningful, and the cancer-specific genes of this network played a role as members of complex biological processes.

## Methods

To build a semi-supervised learning classifier, we first integrated gene expression data with PPI and identified informative gene pairs with the labeled samples. Second, we constructed a sample based graph model using selected informative genes in order to build a classifier.

### Data Description

We downloaded the gene expression datasets of three cancers from the Gene Expression Omnibus (GEO) database. Table 1 summarizes the detailed specification of the datasets. The gene expression dataset GSE2990 was composed of 125 invasive breast cancer samples classified into two groups, high and low risk of recurrence; 64 samples did not have a class label. The gene expression dataset GSE17536 was composed of 177 colorectal cancer patients. Samples were classified into three groups: ‘recurrence,’ ‘no recurrence,’ and ‘unlabeled.’ Based on observation of recurrence within five years of follow-up, the labels were assigned to samples. The unlabeled samples had no clinical follow-up data. The gene expression dataset GSE17538 was composed of 213 colon cancer samples, which were also classified into the three groups mentioned above. A more detailed description of the datasets according to the experimental platform is shown in Table S2 in File S1.

**Table 1:** Datasets used throughout the manuscript.

Cancer type	GEO assess number	No. of labeled samples^1^	No. of unlabeled samples	No. of genes after filtering
Breast	GSE2990	125 (76: −1, 49: +1)	64	13,046
Colorectal	GSE17536	145 (109: −1, 36: +1)	32	13,046
Colon	GSE17538	181 (132: −1, 49: +1)	32	13,046
Breast	GSE4922	249 (160: −1, 89: +1)	0	13,046
Colorectal	GSE18105	111 (67: −1, 44: +1)	0	13,046
**Name**	**Description**	**Quantity**	**Reference**	
Protein-Protein Interaction	Human PPI	108,544(mapped to a gene symbol)	I2D database	

−1: non-recurrence, +1: recurrence.

We also downloaded 194,988 human PPIs from the I2D database, which included known, experimental, and predicted PPIs. Because the proteins in these PPIs were mapped into gene symbols using Universal Protein Resource (UniPROT), we obtained 108,544 PPIs after removing duplicated PPIs and PPIs that contained proteins that were not mapped to a gene symbol.

### System Overview

This section describes a novel graph-based semi-supervised learning algorithm for cancer prognosis. The graph consists of nodes and edges corresponding to samples and interactions between two samples, respectively. The graph is constructed with both labeled and unlabeled samples of gene expression data, and the unlabeled samples were subsequently labeled based on the geometry of the graph structure. Therefore, it is very important to generate a sample-based graph from the given dataset. We propose a novel graph construction method that is specialized for a microarray dataset. Based on this graph construction method, we developed a semi-supervised learning algorithm that uses graph regularization.

In this approach, the graph itself is a classifier. Thus, the parameters for constructing the graph imply that they are the key factors of the classifier. The classification results are dependent on the parameters. Semi-supervised learning generally utilizes the feature or underlying information of unlabeled data. This approach assumes that unlabeled data is able to enhance the classification performance. According to this distinguishing feature of semi-supervised learning, we take advantage of unlabeled data for building a classifier.

**Figure 1:**
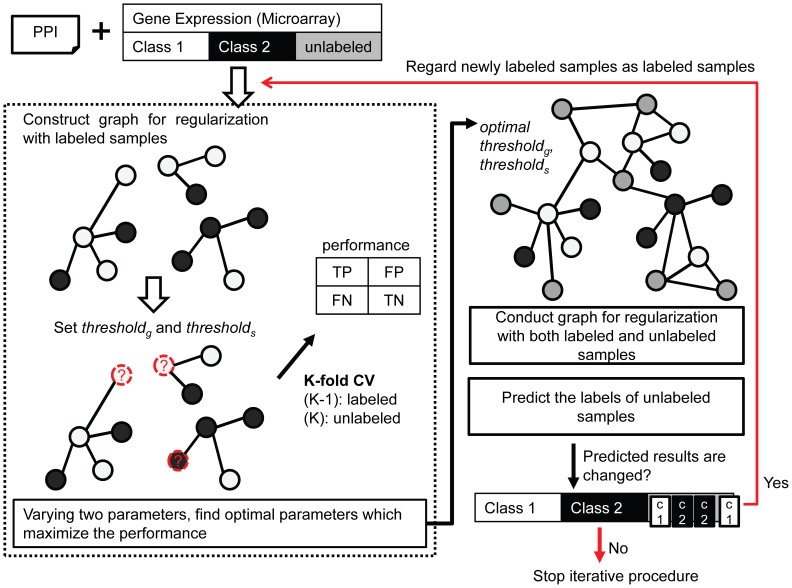
Detailed workflow to determine the optimal parameter set. First, we construct a graph for regularization with only labeled samples by varying two parameters. In this phase, we use *k*-fold cross validation to determine the optimal parameter set. We then apply semi-supervised learning with the obtained optimal parameter set and predict the labels of the unknown samples. The proposed method uses unlabeled sample information to build a classifier by iterating the procedure.

The proposed method has two phases. The first phase is to determine the candidate optimal parameters for graph regularization varying the parameter ranges in *k*-fold cross validation. After this phase, we construct the graph with both labeled and unlabeled samples. Then, we identify whether the classification results from graph regularization are changed or converged. If they are changed, we regard the classified unlabeled data as newly labeled data and use them to determine the optimal candidate parameters. In this iterative process, the information of unlabeled samples is provided. The previous semi-supervised learning method proposed in [9] also used unlabeled samples to build a classifier based on Low Density Separation (LDS). Figure 1 shows the entire workflow including the semi-supervised learning module for determining the optimal parameters of our method.

**Figure 2:**
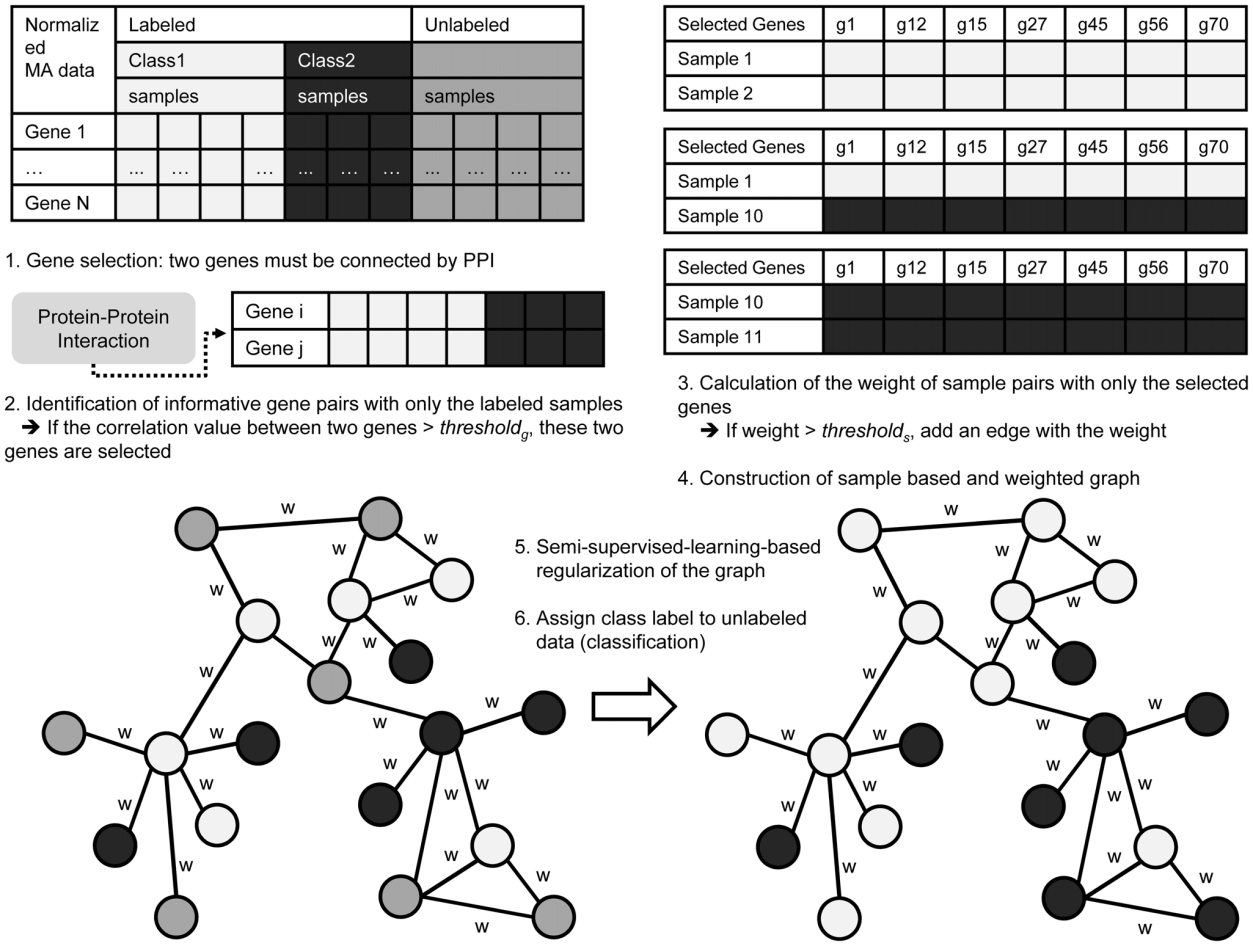
Detailed workflow of the proposed semi-supervised learning algorithm. We apply a graph regularization approach for semi-supervised learning, and the purpose of the proposed method is to predict the labels of unlabeled samples.

The details of the semi-supervised learning module in this workflow are described in the following sections. This module consists of the following three core steps: (1) identification of informative gene pairs, (2) construction of sample graphs with selected genes, and (3) regularization of the graph and prediction of the labels of the unlabeled samples. The workflow of the semi-supervised learning module is shown in Figure 2.

### Identification of Informative Gene Pairs

There are tens of thousands of genes in microarray datasets, and only some of them are specific to the classification of the sample. Informative gene pairs indicate interactions that are diacritical in the two contrary classes of labeled samples. We adopted and modified our previously proposed scheme for identifying interactions in the gene expression dataset [6]. In that study, we demonstrated that the intensity of some interactions can be different between normal cells and tumor cells. We also elucidated that changes in the interaction level could be the cause or effect of tumorigenesis, and that the modification of protein complexes could affect various interactions as a result of tumorigenesis.

The measurement of changes in interactions can be regarded as identification of the degree of dependency between two genes. A large correlation value between two genes as a degree of change indicates that there is strong dependency between the two genes. Based on this rationale, we propose a scoring scheme to calculate the strength of the connection between two genes that are connected by PPIs. Using this measure, we can facilitate the selection of informative interactions from gene expression datasets, since the cancer specific network was constructed based on a similar scoring function. In other words, we can choose the interactions specified for tumor recurrence using the proposed scoring scheme. The score of two genes is calculated by the following equation:
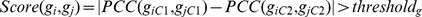
where *g_iC_*_1_ and *g_iC_*_2_ are vectors of the mRNA expression value of gene *i* on class 1 and class 2 samples, respectively, and *g_jC_*_1_ and *g_jC_*_2_ are vectors of the mRNA expression value of gene *j* on class 1 and class 2 samples. Only the gene pairs with a scoring value greater than *threshold_g_* are regarded as being significantly different between two classes. This scoring scheme is performed only with the labeled samples in the gene expression dataset. A simple example of calculating Score values is shown in Figure S1 in File S1.

### Construction of the Sample-based Graph

We constructed a sample-based graph for regularization. The weight of a sample pair is calculated by the Pearson Correlation Coefficient (PCC) between two sample vectors that are composed of the genes as elements, where the genes are obtained from informative gene pairs. Both labeled and unlabeled samples are used in the graph. The weight function is as follows:
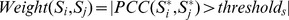
where S^*^_i_ and S^*^_j_ are vectors of the mRNA expression value of sample *i* and sample *j*, respectively, of the selected gene pairs with values larger than *threshold_s_*. We assume that there is a significant relationship between two samples when they are highly associated with each other with a positive or negative pattern. We can transform the gene expression dataset into a graph structure that can be regularized. A simple example of calculation of the Weight value is shown in Figure S1 in File S1.

### Regularization of the Graph

Based on the sample-based graph structure derived from the method mentioned above, labels are assigned to the unlabeled nodes. To achieve this, we employ a basic regularization approach. For the regularization of the graph, we estimate a regularization framework based on the manifold assumptions. The cost function for regularization is as follows:
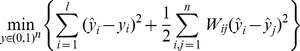
where *y* and *ŷ* respectively indicate the initial labels and the estimated labels for both labeled and unlabeled data. *W_ij_* indicates the weight between node *i* and node *j*. The total number of both labeled and unlabeled nodes is *n*, and the number of labeled nodes is *l*. In our problem, *y* indicates labeled and unlabeled samples of the cancer dataset, and *W_ij_* is obtained using the weight function defined in the above chapter. Using the cost function, we measure the consistency with the initial labeling using the first term, and we assign a penalty for regularization using the second term. Using the second term, we calculate the weighted difference between two nodes without consideration of whether or not they are labeled. The major purpose of this cost function is to minimize the weighted difference among all nodes. This process refers to regularization and is equivalent to the label propagation algorithm. In our case, it is unnecessary to reassign labels to the labeled data because they have already been clinically verified. Therefore, in the first term of the cost function, *ŷ_i_* is constrained to be equal to *y_i_*. As a result, the cost function can be transformed into the following function with a graph Laplacian.



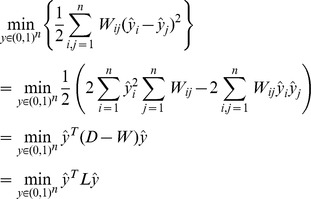
where *L* is the un-normalized graph Laplacian and *D* is a diagonal matrix of weight matrix *W*. This function penalizes rapid label changes in *ŷ* between two close data points according to the given weight matrix. Various approximations have been proposed to minimize this function over *ŷ_u_*, where *ŷ_u_* indicates the estimated label for unlabeled data and *ŷ_l_* indicates the labeled data. Minimizing the function with respect to *ŷ_u_* converts it into the following function.



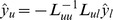



We predict the labels for the unlabeled data using this calculation. Since we do not focus on development of novel semi-supervised learning algorithm, we employ a general regularization approach for the weighted sample graph, and it is sufficient to apply the general approach to our problem.

## Results

We performed experiments to obtain the optimal combination of two thresholds for the score of a gene pair and the weight of the sample based graph. We then compared our method with several existing methods in order to assess its performance. Finally, we analyzed the network derived from our method with the known cancer related gene list.

### Obtaining the Optimal Parameters

We used two parameters to both identify informative gene pairs and assign weights to sample pairs. To find optimal combinations of these two parameters, we measured the accuracy of the proposed classification model using *k*-fold cross validation by varying these two parameters. We changed the *threshold_g_* value from 0.15 to 0.6 in intervals of 0.05 and the *threshold_s_* value from 0.72 to 0.9 in intervals of 0.02. Overall, we performed 100 different experiments, varying these two thresholds and measuring the accuracy of each experiment by averaging the *k* accuracies generated during *k*-fold cross validation. Figure S2 in File S1 depicts the workflow of the evaluation of our method. To measure the accuracy of the semi-supervised learning method, we only used labeled samples and assumed that some of the samples were unlabeled. Using these two groups of labeled and unlabeled samples, we constructed the graph and performed regularization.

To determine the classification of unlabeled samples, we applied a heuristic method called Class Mass Normalization (CMN) proposed by [15]. In general, the decision rule assigns label 1 to node *i* if the calculated value after regularization is greater than 0.5, and label 0 otherwise. However, this decision approach is only effective when the classes are well separated. Since gene expression data do not always have the same number of samples for each class, we adopted CMN to identify the final class label. CMN adjusts the criterion for determining the class label according to the ratio of the mass of classes.

**Figure 3:**
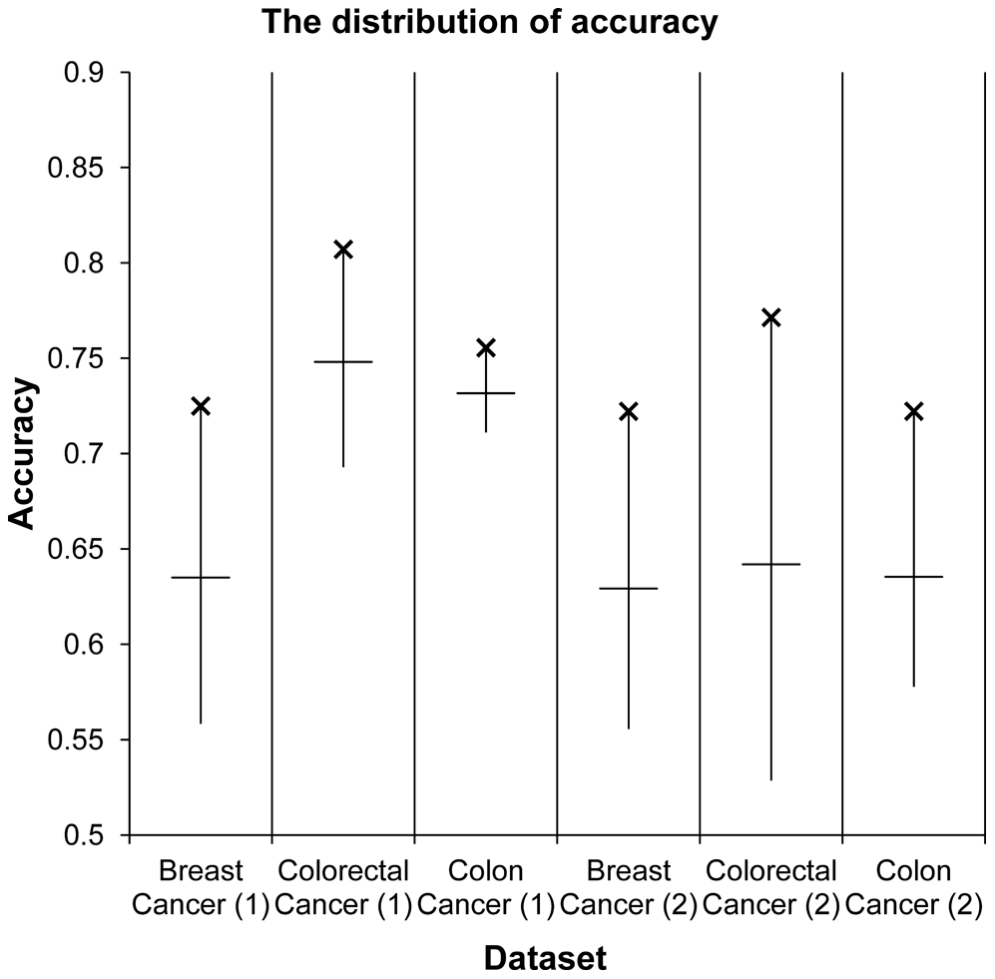
Experimental results of parameter testing. We performed 100 different experiments while changing two threshold values and obtained 100 average accuracies for each dataset using 10-fold cross validation. We found the maximum, minimum, and average accuracies for each dataset in two cases. (1) We carried out 10-fold cross validation over 100 times, varying the two thresholds of the original samples as shown in Table 1. (2) We also carried out 10-fold cross validation over 100 times, varying the two thresholds after balancing the number of samples in the two classes. We randomly removed samples 27, 73, and 83 from the non-recurrence groups GSE2990, GSE17536, and GSE17538, respectively.

The experimental results obtained from varying parameters are shown in Figure 3. We performed 100 different experiments, varying the two threshold values for each dataset. For each experiment, we performed *k*-fold cross validation and averaged the *k* accuracies. The purpose of this process was to compare the accuracy of classification on 100 different experiments. We also carried out the same experiments with an adjusted dataset, which had the same number of samples for both recurrence and non-recurrence groups since different proportions of class labels can affect the performance of the classifier. Our method uses semi-supervised learning-based graph regularization, which is influenced by the geometric structure of the graph, to classify the label. If the relative ratios of two classes differ considerably, the labels of a small number of samples may not be propagated through the graph. This can affect classification performance. All of the chosen cancer datasets were divided into original and adjusted sample groups. In the remainder of this article, we describe an experiment conducted with these two groups. We obtained two optimal threshold values at maximal accuracy for each dataset, as shown in Figure 3. We also found the optimal thresholds while changing the *k* value of cross validation. The experimental results of *k* = 5 and *k* = 20 are described in Table S5 in File S1. The experimental results are shown in Table 2. To show an effectiveness of unlabeled data, we also performed out the experiments varying the number of unlabeled samples. The experimental result substantiated that the accuracy was improved according to increasing of the number of unlabeled samples. This experimental result is shown in table S6 in File S1.

**Table 2:** Optimal combination of two thresholds for each dataset in 10-fold cross validation.

Cross validation	Group	Dataset (# of samplesfor each class)	Optimal *threshold_g_* value		Optimal *threshold_s_* value		Best accuracy	Sen.	Spec.
K = 10	Original	GSE2990 (76: −1, 49: +1, 64: U)		0.20		0.72	0.725	0.617	0.795
		GSE17536 (109: −1, 36: +1, 32: U)		0.15		0.86	0.807	0.485	0.906
		GSE17538 (132: −1, 49: +1, 32: U)		0.20		0.72	0.756	0.163	0.977
	Adjusted	GSE2990 (49: −1, 49: +1, 64: U)		0.45		0.76	0.767	0.721	0.809
		GSE17536 (36: −1, 36: +1, 32: U)		0.15		0.84	0.786	0.882	0.694
		GSE17538 (49: −1, 49: +1, 32: U)		0.35		0.90	0.767	0.756	0.778

Sen. = Sensitivity, Spec. = Specificity.

### Comparison with Existing Methods

We compared the proposed method with three typical supervised classification algorithms implemented in Weka 3.6.8, namely Support Vector Machine (SVM) [16], Naïve Bayesian [17], and Random Forest [18]. In addition, we also compared our method with TSVM, which is a semi-supervised learning version of SVM and was implemented in SVM-light.

We compared the accuracies, including the sensitivities and specificities, of the proposed method and other methods using 10-fold cross validation. We divided the dataset into two groups as mentioned above, and repeated the experiment 15 times each for three cancer types. We calculated the average values of accuracy, sensitivity, and specificity for each dataset in the adjusted group. The sensitivity and specificity of TSVM could not to be calculated since TSVM of SVM-light provided accuracy, precision, and recall. Table 3 summarizes the result of these tests. In the original group, the accuracy of our method was generally better than that of the comparative methods. In particular, the performance difference between the proposed method and other algorithms in the adjusted group was larger than in the original group. If the proportion of class labels is biased in a training dataset, the classifier can be over-fitted toward a larger label. The proportion of class labels in the original group was biased toward the non-recurrence label, “−1.” Therefore, the sensitivity and specificity of most of the methods compared, including our method, were different. Since predicting both labels is important in predicting the recurrence of cancer, higher classification sensitivity and specificity are better. In the adjusted group, our method had higher sensitivity, specificity, and accuracy than the comparison methods. Generally, we confirmed that the proposed method had performance superior to that of the other methods.

**Table 3:** Predicting performance comparison of the proposed method with four existing methods using PPI data to identify informative genes.

Cancer type (GSE No.)	Data description	Proposed method	TSVM	SVM		Naïve Bayesian	Random Forest
	**Original**	**Accuracy (Sensitivity/Specificity)**					
Breast (GSE2990)	L:125(−1∶76, +1∶49) U:64	0.725 (0.617/0.795)	0.543 (−/−)		0.528 (0.671/0.306)	0.592 (0.605/0.571)	0.664 (0.921/0.265)
Colorectal (GSE17536)	L:145(−1∶109, +1∶36) U:32	0.807 (0.485/0.906)	0.752 (−/−)		0.772 (0.889/0.389)	0759 (0.844/0.500)	0.752 (0.963/0.111)
Colon (GSE17538)	L:181(−1∶132, +1∶49) U:32	0.756 (0.163/0.977)	0.728 (−/−)		0.796 (0.917/0.469)	0.707 (0.826/0.388)	0.713 (0.955/0.061)
	**Adjusted**	**Accuracy (Sensitivity/Specificity)**					
Breast (GSE2990)	L:98(−1∶49, +1∶49) U:64	0.767 (0.721/0.809)	0.499 (−/−)		0.510 (0.495/0.525)	0.576 (0.574/0.565)	0.522 (0.418/0.627)
Colorectal (GSE17536)	L:72(−1∶36, +1∶36) U:32	0.786 (0.882/0.694)	0.499 (−/−)		0.630 (0.672/0.587)	0.640 (0.628/0.652)	0.597 (0.550/0.644)
Colon (GSE17538)	L:98(−1∶49, +1∶49) U:32	0.767 (0.756/0.778)	0.498 (−/−)		0.635 (0.657/0.614)	0.592 (0.465/0.718)	0.572 (0.486/0.663)

For each experiment, the optimal combination of two thresholds was obtained using the approach mentioned above and was applied to an independent test using unlabeled samples. Bold font indicates the superior performer.

TSVM: *P* (the ratio of two class labels).

SVM: PolyKernel –C 250007–E 1.0, The complexity parameter C (1.0), epsilon (1.0E−12), filterType (Normalized training data).

Naïve Bayesian: No parameters.

Random Forest: numTrees (10), seed (1).

**Figure 4:**
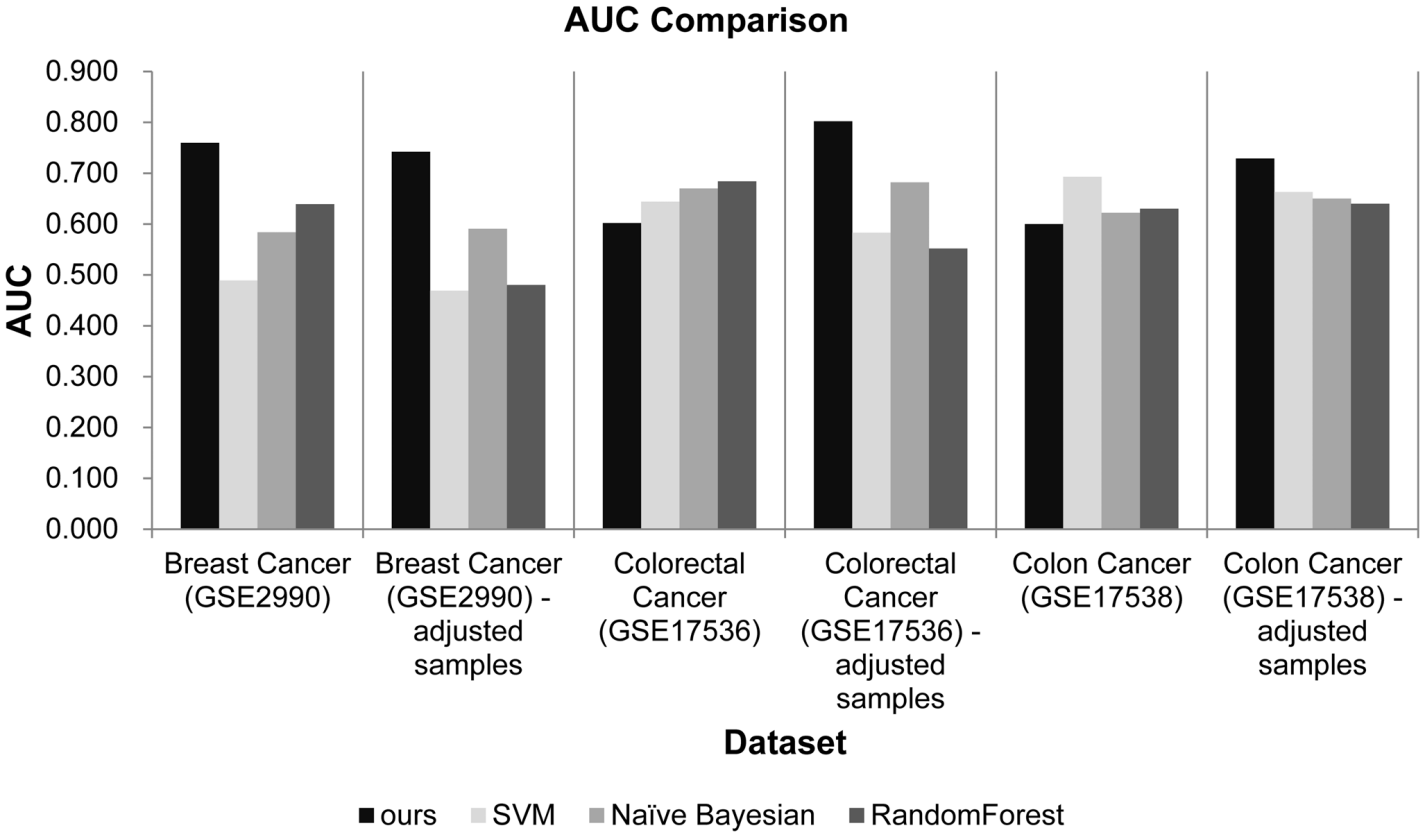
Experimental results of AUC comparison of the proposed method with three existing methods. We compared AUC values of the proposed method and other supervised learning algorithms.

The average accuracy increased 24.9% compared to the four existing methods. For example, as shown in Table 3, the accuracy of the proposed method was 0.725 and the accuracy of TSVM was 0.543 for the breast cancer dataset without adjusting the class label ratio, an approximate 33% improvement. The average improvement ratio for all datasets was 24.9%. Five of six experimental datasets included the adjusted sample groups, and the accuracy of the proposed method was higher than the existing methods. The average difference in accuracy of the proposed method and its competitors was 0.139. We also obtained AUC values for each experimental dataset. As shown in Figure 4, the proposed method showed a particularly higher AUC value for the breast cancer dataset and a higher AUC value compared to other existing methods for four of the six experimental datasets.

In addition, we performed an independent test where we applied relief-F to select informative genes instead of PPI. We also carried out a statistical analysis of significant difference in accuracy for comparison among methods. The detailed experimental results are described in the supporting information of Table S1, Table S3, and Table S4 in File S1.

## Discussion

The performance of a classification method is influenced by the proportion of training data in each class. The computational contribution of the proposed method is determination of the coherent accuracy of the differences in class proportion. This is advantageous since the number of samples for each class cannot be adjusted during independent testing. In addition, though classification based on semi-supervised learning has been applied to microarray datasets, the results of the proposed method demonstrate that the approach based on the ‘smoothness assumption’ was sufficient for clinical application.

**Figure 5:**
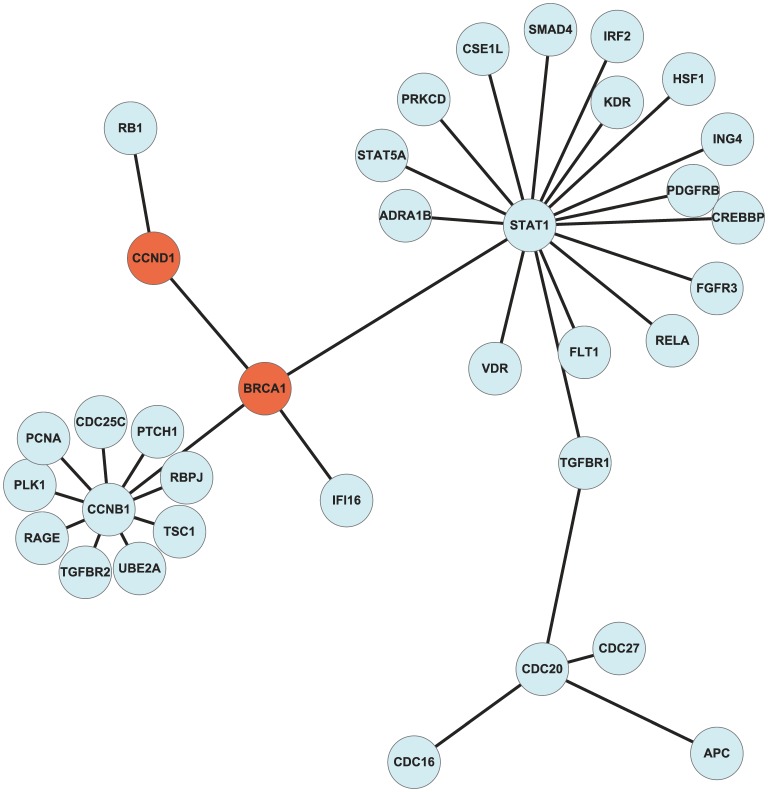
Representation of a breast cancer recurrence-specific gene sub-network related to cancer proliferation. The orange-colored nodes are oncogenes.

To reduce the dimension of the microarray data, we selected gene sets with strong biological interactions. Therefore, the sample-based graph of regularization was constructed based on biological knowledge. The selected gene set can be referred to as a recurrence-specific gene network. Our analysis demonstrated that this gene network was biologically meaningful in regard to cancer recurrence. To analyze the cancer-recurrence-specific gene network, we enriched the informative gene set derived from the optimal parameter set using the Gene Ontology (GO) database and BiNGO [19]. Among the many enriched GO terms, we focused on those related to cancer recurrence. Among several recurrence related terms, we focused on GO terms related to “proliferation” and analyzed the sub-gene networks for those GO terms, referring to the literature. To better analyze the details of the sub networks related to proliferation in each cancer, we illustrated the networks using Cytoscape [20], as shown in Figure 5, Figure S3 in File S1, and Figure S4 in File S1.

The proposed method identified the sub-gene network composed of BRCA1, CCND1, STAT1, and CCNB1, shown in Figure 4, where the primary oncogene BRCA1 was connected with another oncogene CCND1 and two hub-structured genes, CCNB1 and STAT1. We assumed that these gene sub-networks were related to breast cancer recurrence. The CCND1, CCNB1, and STAT1 genes neighboring BRCA1 have also been reported to have important roles in breast cancer recurrence. CCND1 is a primary gene in the regulation of cell cycle progression, and Shu *et al*. reported an association between breast cancer risk and survival based on CCND1 polymorphisms [21]. CCNB1 an oncotype DX gene was reported that STAT1 was significantly related to the activation of IFN-γ and its antitumor effects [22], [23]. If the STAT1-dependent expression of MHC proteins is enhanced, tumor proliferation and survival are inhibited by the activation of IFN-γ. Desmedt *et al*. concluded that activation of STAT1 plays an important role in the death of tumor cells and the activation of apoptotic genes [23].

## Conclusions

In this study, we proposed a novel semi-supervised learning method based on graph regularization in order to predict cancer recurrence. We also showed that the recurrence-specific gene networks derived from the proposed method contain many recurrence-related genes. We integrated the PPI data with the gene expression data to produce an informative gene set and to analyze the biological process related to recurrence. We also used a graph regularization approach and semi-supervised learning methods to predict the labels of unknown samples. We confirmed that the performance of the proposed method was better than that of several preexisting classification methods for colorectal and colon datasets, which had the same proportion of class labels. In the case of the breast cancer dataset, the proposed method showed outstanding performance compared to the existing method with both the original and the adjusted samples. Also, the proposed method was superior to TSVM for three cancer datasets. It is necessary to utilize the unlabeled samples because labeling medically many samples is time consuming and expensive. In a medical dataset, however, it is difficult to obtain a dataset with a balanced number of samples. We plan to focus on solving this problem of the semi-supervised learning-based method in future work. Last, we identified the functional relationships among recurrence related genes by constructing gene networks. We concluded that the proposed method, which uses many data points without class labels, is suitable for prognosis prediction and analysis of the biological roles of genes related to cancer recurrence.

## Supporting Information

File S1 **File S1 contains four supplemental figures and, five supplemental tables.**
(DOC)
