# Supplementary material for: Integrative Gene Network Construction to Analyze Cancer Recurrence Using Semi-Supervised Learning
Source: PLoS One. 2014 Jan 31;9(1):e86309. doi: 10.1371/journal.pone.0086309 (PMC3908883; doi:10.1371/journal.pone.0086309)
Supplement: File S1 — contains four supplemental figures and, five supplemental tables. (DOC) [file pone.0086309.s001.doc]

**[Additional File S1]**

**Integrative Gene Network Construction to Analyze Cancer Recurrence using Semi-Supervised Learning**

**Chihyun Park^1^, Jaegyoon Ahn ^1^, Hyunjin Kim^1^, Sanghyun Park^1§^**

^1^Department of Computer Science, Yonsei University, South Korea

**Supplementary information contents**

1. **An example of calculating Score values ……………………. 2**
2. **Workflow of the evaluation for the proposed method ……………………………………. 3**
3. **Additional experimental results ……………………………………….. 4**
4. **More detailed analysis of gene-sub networks of colorectal and colon cancer. ………… 9**

^§^To whom correspondence should be addressed.

**1. An example of calculating *Score* values**


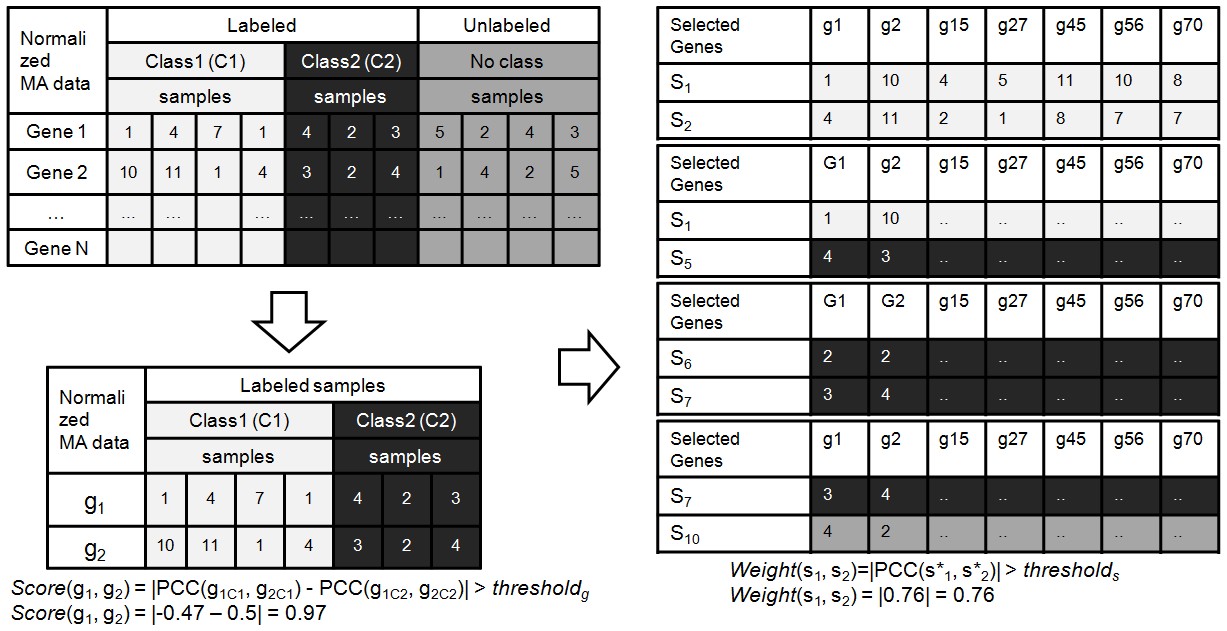


Figure S1. Example of calculating Score and Weight values. The left and the right examples show the calculation of Score and Weight values, respectively.

**2. Workflow of the evaluation for the proposed method**


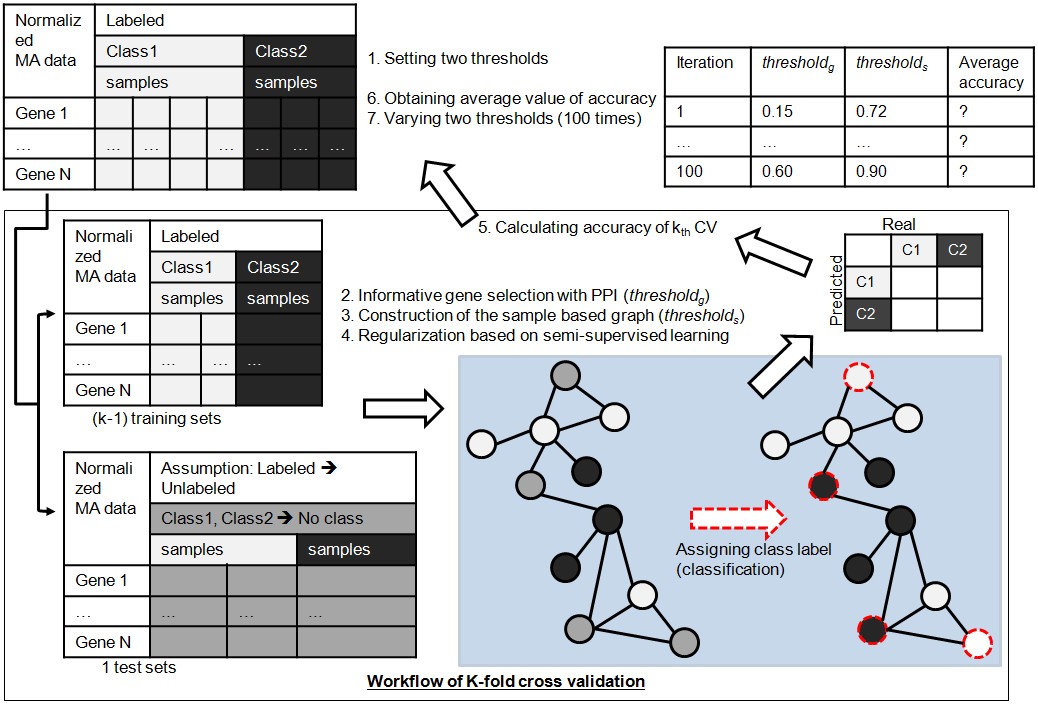


Figure S2. Workflow of the evaluation for the proposed method. We measured the accuracy, sensitivity, and specificity of each candidate parameter set using *k*-fold cross validation and chose two optimal parameters.

**3. Additional experimental results**

We performed independent tests on breast cancer. In order to measure the performance of the proposed method using an independent dataset, we used one breast cancer datasets, GSE2990, for determining the learning classifier and the other, GSE4922, for an independent test. We measured accuracy, sensitivity, and specificity using the two optimal parameters mentioned above. We also compared the performance of the independent test to the four existing classification methods described above. The results are shown in Table S1. The performance was generally worse than that obtained from the 10-fold cross validation. We assumed that the difference of microarray platform and the sample set could decrease the classifying performance. Integration of data from different microarrays has been one of the challenges of bioinformatics. The correlation between two samples from different datasets tended to be low. Therefore, even though we normalized the dataset, the graph Laplacian matrix from all samples was clearly separated according to the dataset, and this was the cause of the decrease in performance. The difficulty of independent testing has been previously described in [1]. Shi *et al*. suggested that the cause of this problem was the biased class label proportion and the biological differences between patient cohorts [1]. The basic statistics of the microarray dataset used for the independent test are presented in Table S2. The distribution of expression values varied according to both the microarray platform and the patients.

Table S1. Performance comparison of independent testing using optimal parameters. The values are accuracy, sensitivity, and specificity, in order, as shown in Table 3.

| **Cancer Type** | **Mode** | **Training dataset** | **Test dataset** | **Our method** | **TSVM** | **SVM** | **Naïve Bayesian** | **Random Forest** |
| --- | --- | --- | --- | --- | --- | --- | --- | --- |
| Breast | Original samples | GSE2990 L: 125(-1: 76, +1: 49) | GSE4922 L: 249(-1: 160, +1: 89) | 0.422 (0.416 / 0.425) | 0.642 ( - / - ) | 0.402 (0.955 / 0.094) | 0.643 (0.000 / 1.000) | 0.635 (0.213 / 0.869) |
|  | Adjusted samples | GSE2990 L: 98(-1: 49,+1: 49) | GSE4922 L: 249(-1: 160, +1: 89) | 0.494 (0.404 / 0.544) | 0.639 ( - / - ) | 0.558 (0.764 / 0.444) | 0.643 (0.000 / 1.000) | 0.590 (0.831 / 0.613) |
| Colorectal | Original samples | GSE17536 L: 145(-1: 109,+1: 36) | GSE18105 L: 111(-1: 67, +1: 44) | 0.532 (0.636 / 0.463) | 0.604 ( - / - ) | 0.613 (0.002 / 1.000) | 0.604 (0.000 / 1.000) | 0.604 (0.000 / 1.000) |
|  | Adjusted samples | GSE17536 L: 72(-1: 36,+1: 36) | GSE18105 L: 111(-1: 67, +1: 44) | 0.441 (0.886 / 0.149) | 0.604 ( - / - ) | 0.604 (0.985 / 0.023) | 0.613 (0.022 / 1.000) | 0.622 (0.386 / 0.776) |

TSVM: *P* (the ratio of two class labels)

SVM: PolyKernel –C 250007 –E 1.0, The complexity parameter C (1.0), epsilon (1.0E-12), filterType (Normalize training data)

Naïve Bayesian: No parameters

RandomForest: numTrees (10), seed (1)

Table S2. Features of the microarray data according to the experimental platform.

| **Data type** | **Independent dataset** | **MA platform** | **Normalization** | | **Mean** | **SD** |
| --- | --- | --- | --- | --- | --- | --- |
| Training dataset 1 | **GSE2990** L: 125 (-1: 76, +1: 49) U: 64 | GPL96 | N | 6.948 | | 2.011 |
|  |  |  | Y | -1.32922e-013 | | 1.000 |
| Test dataset 1.1 | **GSE4922** L: 289 (-1: 160 +1: 89) | GPL96 | N | 6.283 | | 1.697 |
|  |  |  | Y | 2.7212e-014 | | 1.000 |
| Test dataset 1.2 | GSE3494 L: 236 (-1: 181, +1: 55) | GPL96 | N | 6.282 | | 1.696 |
|  |  |  | Y | 1.57633e-013 | | 1.000 |
| Test dataset 1.3 | GSE2034 L: 255 (-1: 195, +1: 60) | GPL96 | N | 987.501 | | 3268.990 |
|  |  |  | Y | -3.75389e-014 | | 1.000 |
| Test dataset 1.4 | GSE9893 L: 155 (-1: 103, +1: 52) | GPL5049 | N | 0.508 | | 1.908 |
|  |  |  | Y | -6.38399e-014 | | 1.000 |
| Test dataset 1.5 | GSE33926 L: 51 (-1: 39, +1: 12) | GPL7264 | N | 0.042 | | 1.067 |
|  |  |  | Y | -4.43993e-015 | | 1.000 |
| Training dataset 2 | **GSE17536** L: 145 (-1: 109, +1: 36) U: 32 | GPL570 | N | 8.028 | | 1.862 |
|  |  |  | Y | 1.00412e-013 | | 1.000 |
| Test dataset 2.1 | **GSE18105** L: 111 (-1: 67, +1: 44) | GPL570 | N | 6.939 | | 2.440 |
|  |  |  | Y | 5.23479e-014 | | 1.000 |

We compared the performance of our method to four existing methods using relief-F [2], which is a feature selection method used in place of PPI for identifying informative genes. We confirmed that the proposed method had outstanding accuracy compared with other algorithms. The experimental results are presented in Table S3. Generally, the performance of our method when the PPI data was integrated with the gene expression dataset was slightly better than the performance when relief-F was used for selecting the informative genes. The genes connected by protein interactions tended to be more biologically meaningful than genes chosen by the feature selection method, which only took into account the expression values of the used dataset. In conclusion, our method, which was based on semi-supervised learning, was superior to the other algorithms tested, which used PPI and relief-F, in both cases. Since integrating PPI data with gene expression data was more meaningful than applying relief-F, we proposed a method that uses PPI data.

Table S3. Performance comparison of the proposed method with four existing methods that use relief-F for selecting informative genes. The values reported are accuracy, sensitivity, and specificity, in order, as shown in Table 3. Bold font indicates the superior performer.

| **Cancer Type (GSE No.)** | **Data description** | **No. of genes** | **Ours with PPI** | **Ours with relief-F** | **TSVM** | **SVM** | **Naïve Bayesian** | **Random Forest** |
| --- | --- | --- | --- | --- | --- | --- | --- | --- |
| Breast(GSE2990) | L: 125 (-1: 76, +1: 49) | 10 | **0.725** (0.653/0.774) | **0.692** (0.583/0.764) | 0.593 (- / -) | 0.608 (0.000 / 1.000) | 0.584 (0.592 / 0.579) | 0.632 (0.347 / 0.816) |
|  |  | 50 |  | **0.708** (0.553/0.808) | 0.580 (- / -) | 0.656 (0.327 / 0.868) | 0.584 (0.592 / 0.579) | 0.552 (0.286 / 0.724) |
|  |  | 100 |  | **0.700** (0.500/0.824) | 0.606 (- / -) | 0.592 (0.264 / 0.803) | 0.584 (0.592 / 0.579) | 0.552 (0.204 / 0.776) |
|  | L: 98 (-1: 49, +1: 49) | 10 | **0.724** (0.740/0.706) | **0.689** (0.696/0.682) | 0.503 (- / -) | 0.551 (0.571 / 0.531) | 0.561 (0.592 / 0.531) | 0.500 (0.510 / 0.490) |
|  |  | 50 |  | **0.711** (0.630/0.795) | 0.489 (- / -) | 0.571 (0.551 / 0.592) | 0.561 (0.592 / 0.531) | 0.490 (0.510 / 0.469) |
|  |  | 100 |  | **0.711** (0.771/0.643) | 0.496 (- / -) | 0.429 (0.429 / 0.429) | 0.602 (0.592 / 0.612) | 0.469 (0.367 / 0.571) |
| Colorectal(GSE17536) | L: 145 (-1: 109, +1: 36) | 10 | **0.793** (0.272/0.953) | **0.793** (0.273/0.653) | 0.750 (- / -) | 0.751 (0.000 / 1.000) | 0.662 (0.444 / 0.734) | 0.724 (0.000 / 0.963) |
|  |  | 50 |  | **0.786** (0.265/0.953) | 0.625 (- / -) | 0.759 (0.056 / 0.991) | 0.566 (0.389 / 0.624) | 0.717 (0.056 / 0.936) |
|  |  | 100 |  | **0.807** (0.333/0.953) | 0.683 (- / -) | 0.710 (0.167 / 0.890) | 0.607 (0.528 / 0.633) | 0.710 (0.028 / 0.936) |
|  | L: 72 (-1: 36, +1: 36) | 10 | **0.760** (0.779/0.739) | **0.757** (0.743/0.771) | 0.478 (- / -) | 0.403 (0.389 / 0.417) | 0.556 (0.694 / 0.417) | 0.569 (0.472 / 0.667) |
|  |  | 50 |  | **0.729** (0.765/0.694) | 0.558 (- / -) | 0.583 (0.611 / 0.556) | 0.569 (0.722 / 0.417) | 0.556 (0.472 / 0.639) |
|  |  | 100 |  | **0.729** (0.886/0.571) | 0.480 (- / -) | 0.500 (0.500 / 0.500) | 0.500 (0.528 / 0.472) | 0.500 (0.444 / 0.556) |
| Colon(GSE17538) | L: 181 (-1: 132, +1: 49) | 10 | **0.756** (0.365/0.938) | **0.756** (0.143/0.685) | 0.731 (- / -) | 0.729 (0.000 / 1.000) | 0.669 (0.184 / 0.848) | 0.690 (0.102 / 0.909) |
|  |  | 50 |  | **0.756** (0.167/0.970) | 0.711 (- / -) | 0.729 (0.020 / 0.992) | 0.669 (0.245 / 0.826) | 0.713 (0.102 / 0.939) |
|  |  | 100 |  | **0.783** (0.388/0.931) | 0.671 (- / -) | 0.729 (0.122 / 0.955) | 0.674 (0.254 / 0.833) | 0.718 (0.143 / 0.932) |
|  | L: 98 (-1: 49, +1: 49) | 10 | **0.733** (0.757/0.711) | **0.678** (0.674/0.682) | 0.483 (- / -) | 0.551 (0.347 / 0.755) | 0.541 (0.286 / 0.796) | 0.531 (0.469 / 0.592) |
|  |  | 50 |  | **0.711** (0.705/0.717) | 0.493 (- / -) | 0.480 (0.408 / 0.551) | 0.520 (0.286 / 0.755) | 0.571 (0.571 / 0.571) |
|  |  | 100 |  | **0.744** (0.644/0.844) | 0.508 (- / -) | 0.520 (0.510 / 0.531) | 0.571 (0.347 / 0.796) | 0.520 (0.490 / 0.551) |

TSVM: *P* (the ratio of two class labels)

SVM: PolyKernel –C 250007 –E 1.0, The complexity parameter C (1.0), epsilon (1.0E-12), filterType (Normalize training data)

Naïve Bayesian: No parameters

RandomForest: numTrees (10), seed (1)

Table S4. Statistical analysis of significant difference in accuracy between the proposed method and four existing methods. We carried out paired t-test and applied 0.05 as significance level.

| **Cancer Type (GSE No.)** | **Data description** | **Ours vs. TSVM** | **Ours vs. SVM** | **Ours vs. Naïve Bayesian** | **Ours vs. Random Forest** |
| --- | --- | --- | --- | --- | --- |
|  | **Original** | **Statistics (T-test)** | | | |
| Breast (GSE2990) | L: 125 (-1: 76, +1: 49) U: 64 | 0.0407 | 0.3095 | 0.1080 | 0.0527 |
| Colorectal (GSE17536) | L: 145 (-1: 109, +1: 36) U: 32 | 0.0013 | 0.0394 | 0.5294 | 0.0065 |
| Colon (GSE17538) | L: 181 (-1: 132, +1: 49) U: 32 | 0.0081 | 0.0003 | 0.0103 | 0.0000 |
|  | **Adjusted** | **Statistics (T-test)** | | | |
| Breast (GSE2990) | L: 98 (-1: 49, +1: 49) U: 64 | 0.0037 | 0.8342 | 0.9983 | 0.1821 |
| Colorectal (GSE17536) | L: 72 (-1: 36, +1: 36) U: 32 | 0.0016 | 0.9992 | 0.1213 | 0.4834 |
| Colon (GSE17538) | L: 98 (-1: 49, +1: 49) U: 32 | 0.3629 | 0.0005 | 0.0105 | 0.0005 |

Table S5. Optimal combination of two thresholds for each dataset in varying *k* in *k*-fold cross validation

| **Cross Validation** | **Group** | **Dataset (# of samples for each class)** | **Optimal *Threshold_g_* Value** | | **Optimal *Threshold_s_* Value** | | **Best Accuracy** | **Sen.** | **Spec.** |
| --- | --- | --- | --- | --- | --- | --- | --- | --- | --- |
| K= 5 | Original | GSE2990 (76: -1, 49: +1, 64: U) | | 0.25 | | 0.72 | 0.696 | 0.367 | 0.908 |
|  |  | GSE17536 (109: -1, 36: +1, 32: U) | | 0.5 | | 0.88 | 0.759 | 0.055 | 0.990 |
|  |  | GSE17538 (132: -1, 49: +1, 32: U) | | 0.25 | | 0.80 | 0.733 | 0.000 | 1.000 |
|  | Adjusted | GSE2990 (49: -1, 49: +1, 64: U) | | 0.30 | | 0.76 | 0.695 | 0.792 | 0.596 |
|  |  | GSE17536 (36: -1, 36: +1, 32: U) | | 0.15 | | 0.86 | 0.743 | 0.588 | 0.889 |
|  |  | GSE17538 (49: -1, 49: +1, 32: U) | | 0.35 | | 0.88 | 0.674 | 0.771 | 0.574 |
| K= 20 | Original | GSE2990 (76: -1, 49: +1, 64: U) | | 0.25 | | 0.84 | 0.767 | 0.553 | 0.904 |
|  |  | GSE17536 (109: -1, 36: +1, 32: U) | | 0.15 | | 0.82 | 0.807 | 0.303 | 0.963 |
|  |  | GSE17538 (132: -1, 49: +1, 32: U) | | 0.60 | | 0.72 | 0.772 | 0.167 | 0.992 |
|  | Adjusted | GSE2990 (49: -1, 49: +1, 64: U) | | 0.45 | | 0.82 | 0.800 | 0.789 | 0.810 |
|  |  | GSE17536 (36: -1, 36: +1, 32: U) | | 0.45 | | 0.88 | 0.867 | 0.828 | 0.903 |
|  |  | GSE17538 (49: -1, 49: +1, 32: U) | | 0.45 | | 0.88 | 0.825 | 0.846 | 0.805 |

Sen. = Sensitivity, Spec. = Specificity

Table S6. Performance of the proposed method in breast cancer dataset based on different numbers of unlabeled samples

| **Cross Validation** | **Group** | **Dataset (# of samples for each class)** | **Optimal *threshold_g_* Value** | | **Optimal *threshold_s_* Value** | | **Best Accuracy** | **Sen.** | **Spec.** |
| --- | --- | --- | --- | --- | --- | --- | --- | --- | --- |
| K=10 | Original | GSE2990 (76: -1, 49: +1, 10: U) | | 0.30 | | 0.80 | 0.708 | 0.326 | 0.945 |
|  |  | GSE2990 (76: -1, 49: +1, 20: U) | | 0.30 | | 0.78 | 0.708 | 0.604 | 0.777 |
|  |  | GSE2990 (76: -1, 49: +1, 30: U) | | 0.45 | | 0.74 | 0.708 | 0.308 | 0.917 |
|  |  | GSE2990 (76: -1, 49: +1, 40: U) | | 0.40 | | 0.72 | 0.717 | 0.522 | 0.839 |
|  |  | GSE2990 (76: -1, 49: +1, 50: U) | | 0.60 | | 0.82 | 0.717 | 0.531 | 0.835 |
|  |  | GSE2990 (76: -1, 49: +1, 64: U) | | 0.20 | | 0.72 | 0.725 | 0.617 | 0.795 |

Sen. = Sensitivity, Spec. = Specificity

**4. More detailed analysis of gene-sub networks of colorectal and colon cancer**


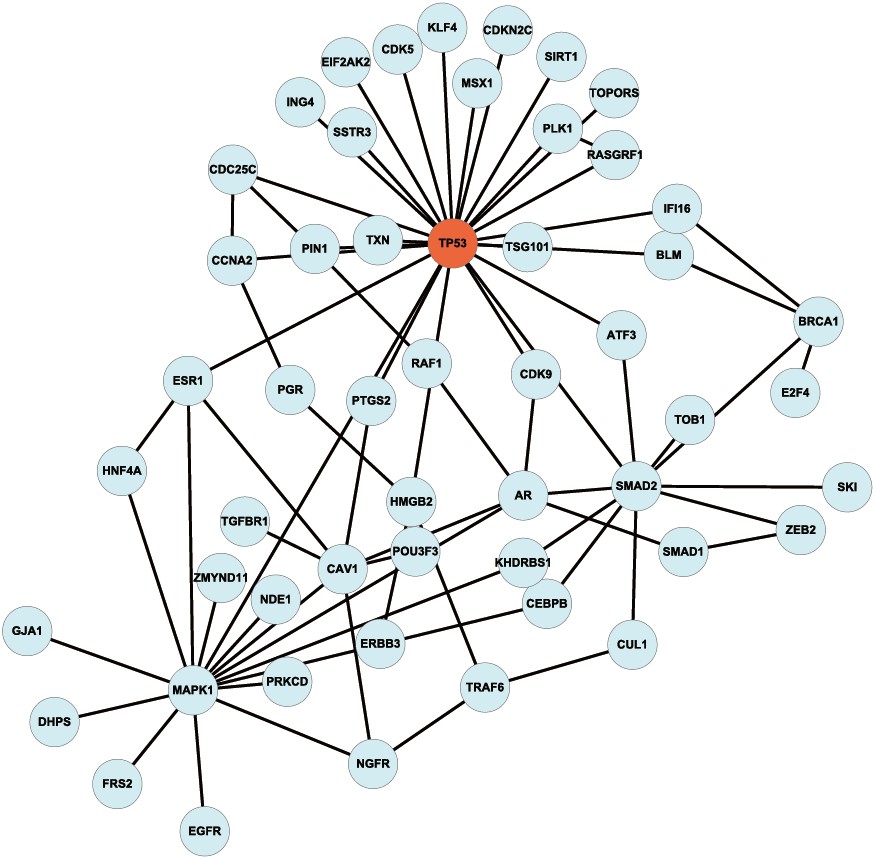


Figure S3. Representation of colorectal cancer recurrence-specific gene sub-network related to proliferation. The orange-colored nodes are oncogenes.

The proposed method identified the sub-gene network including TP53, MAPK1, SMAD2, PIN1, shown in Figure S3, where the primary oncogene TP53 was connected with two hub-structured genes: MAPK1 and SMAD2. The genes neighboring TP53 have also been reported to have an important role in colorectal cancer recurrence. Li *et al*. reported that MAPK1 is related to the chemokine signaling pathway, which is significantly associated with tumor recurrence [3]. SMAD4 is in the same family as SMAD2 and is a known tumor suppressor gene that mediates transforming growth factor-β superfamily signaling. The genetic loss of the location that includes SMAD4 is common in Duke colorectal tumors. Alazzouzi *et al*. demonstrated that patients who had highly expressed SMAD4 had better disease-free survival than those who did not. PIN1 is in the Wnt pathway, which controls cell-to-cell communication regarding processes such as cell proliferation and differentiation [4]. Fang *et al*. used key factors of the Wnt pathway, including PIN1, as biomarkers for colorectal cancer recurrence [5].


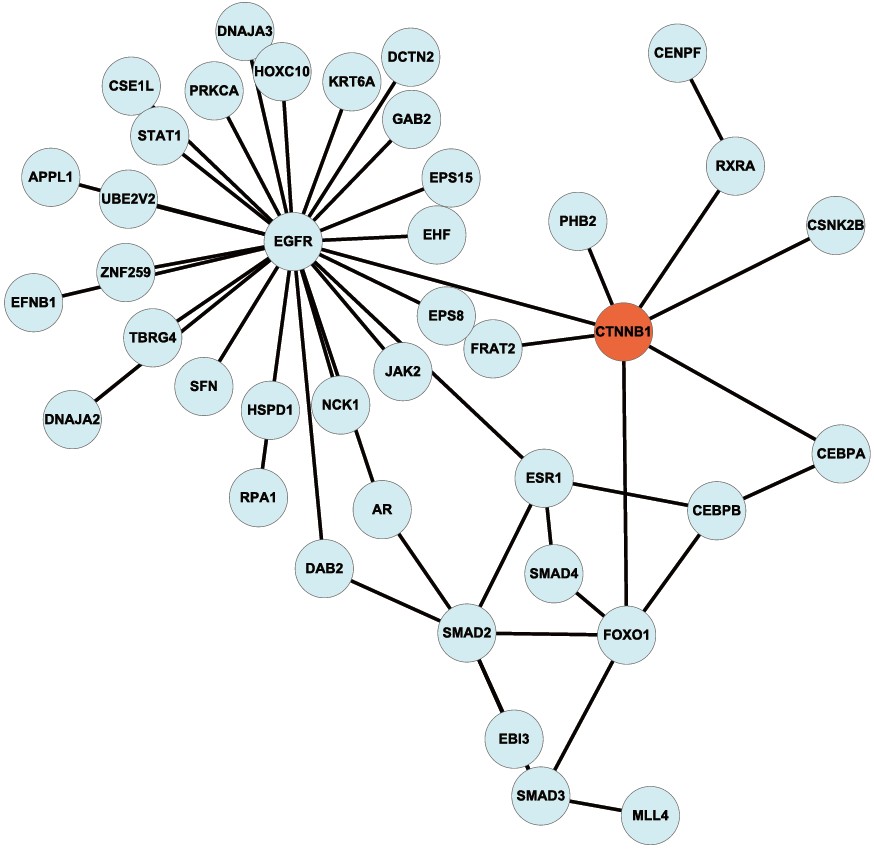


Figure S4. Representation of the colon cancer recurrence-specific gene sub-network related to proliferation. The orange-colored nodes are oncogenes.

The proposed method identified the sub-gene network including CTNNB1 and EGFR, shown in Figure S4, where the primary oncogene CTNNB1 was connected with one hub-structured gene: EGFR. The gene neighboring CTNNB1 also has been reported to have an important role in colon cancer recurrence. Galizia *et al* reported that EGFR expression was associated with disease recurrence and decreased survival by analyzing 126 patients with curatively resected colon cancers [6]. They concluded that anti-EGFR drugs can act as an adjuvant treatment for patients with EGFR-positive colon cancer. Rensnick *et al*. also found that increased p53 expression and strong membranous EGFR expression were significantly associated with colon cancer recurrence and decreased survival [7].

References

1. Shi M., Zhang B. (2011) Semi-supervised learning improves gene expression based prediction of cancer recurrence. Bioinformatics 27: 3017-3023.
2. Kira K. and Rendell L.L. (1992) A practical approach to feature selection. In Proceedings of 9th International Workshop on Machine learning, Morgan Kaufmann Publishers Inc. 249–256.
3. Li W., Wang R., Yan Z., Bai L., Sun Z. (2012) High Accordance in Prognosis Prediction of Colorectal Cancer across Independent Datasets by Multi-Gene Module Expression Profiles. PLoS One 7: e33653.
4. Alazzouzi H., Alhopuro P., Salovaara R., Sammalkorpi H., Järvinen H. (2005) SMAD4 as a Prognostic Marker in Colorectal Cancer. Clin. Cancer Res. 11: 2606-2611.
5. Fang YJ., Lu ZH., Wang GQ., Pan ZZ., Zhou ZW. et al. (2009) Elevated expressions of MMP7, TROP2, and surviving are associated with survival, disease recurrence, and liver metastasis of colon cancer. Int. J. Colorectal Dis. 24: 875-884.
6. Galizia G., Lieto E., Ferraraccio F., De Vita F., Castellano P. et al. (2006) Prognostic Signiﬁcance of Epidermal Growth Factor Receptor Expression in Colon Cancer Patients Undergoing Curative Surgery. Ann. Surg. Oncol. 13: 823-835.
7. Rensnick MB. Routhier J., Konkin T., Sabo E., Pricolo VE. (2005) Epidermal Growth Factor Receptor, c-MET, β-Catenin, and p53 Expression as Prognostic Indicators in Stage II Colon Cancer A Tissue Microarray Study. Clin Cancer Res. 10: 3069-3075.
